# Supplementary material for: Prevalence of symptomatic dry eye disease with associated risk factors among medical students at Chiang Mai University due to increased screen time and stress during COVID-19 pandemic
Source: PLoS One. 2022 Mar 23;17(3):e0265733. doi: 10.1371/journal.pone.0265733 (PMC8942203; doi:10.1371/journal.pone.0265733)
Supplement: S1 File — (PDF) [file pone.0265733.s001.pdf]

Questionnaire for demographic data and risk factors for DED

1. Academic year ☐ 1st -year ☐ 2nd -year ☐ 3th -year
   ☐ 4th -year ☐ 5th -year ☐ 6th -year
2. Age ______ year-old
3. Gender ☐ Male ☐ Female
4. Do you have nearsighted or myopia? ☐ Yes ☐ No
5. Do you wear glasses for correct distance vision? ☐ Yes ☐ No
6. Do you use contact lenses? ☐ Yes ☐ No
7. If you use contact lenses, what type of contact lenses do you use?
   ☐ Daily ☐ Monthly ☐ Yearly
8. Length of time to wear contact lenses _________ hours per day.
9. How many times that you use artificial tears eye drop? ______ times per day.
10. Have you ever had laser vision correction (such as LASIK or PRK surgery)?
    ☐ Yes ☐ No
11. The number of hours that you use your computer / tablet / mobile phone ________hours per day.
12. The number of hours that you read a book _______ hours per day.
