# Supplementary material for: Prevalence of symptomatic dry eye disease with associated risk factors among medical students at Chiang Mai University due to increased screen time and stress during COVID-19 pandemic
Source: PLoS One. 2022 Mar 23;17(3):e0265733. doi: 10.1371/journal.pone.0265733 (PMC8942203; doi:10.1371/journal.pone.0265733)
Supplement: S2 File — (PDF) [file pone.0265733.s002.pdf]

# แบบสอบถาม

**ส่วนที่ 1,2: ข้อมูลทั่วไปและปัจจัยเสี่ยงต่าง ๆของอาการตาแห้ง**

1. ชั้นปีการศึกษา ☐ ปีที่ 1 ☐ ปีที่ 2 ☐ ปีที่ 3

   ☐ ปีที่ 4 ☐ ปีที่ 5 ☐ ปีที่ 6

2. อายุ ______ ปี

3. ท่านมีภาวะสายตาสั้นหรือไม่ ☐ มี ☐ ไม่มี

4. ท่านสวมแว่นสายตาหรือไม่ ☐ มี ☐ ไม่มี

5. ท่านใช้คอนแทคเลนส์หรือไม่ ☐ มี ☐ ไม่มี

6. หากท่านใช้คอนแทคเลนส์ ชนิดของคอนแทคเลนส์ที่ท่านใช้

   ☐ รายวัน ☐ รายเดือน ☐ รายปี

7. ระยะเวลาในการใส่คอนแทคเลนส์ _______ ชั่วโมงต่อวัน

8. ท่านใช้น้ำตาเทียมวันละ ______ ครั้ง

9. ท่านเคยได้รับการผ่าตัดเลสิกหรือไม่

   ☐ เคย ☐ ไม่เคย

10. จำนวนชั่วโมงที่ท่านใช้คอมพิวเตอร์ / แท็บเล็ต/ มือถือ ________ ชั่วโมงต่อวัน

11. จำนวนชั่วโมงที่ท่านอ่านหนังสือเล่ม _______ เล่มต่อวัน
