# Supplementary material for: Prevalence of symptomatic dry eye disease with associated risk factors among medical students at Chiang Mai University due to increased screen time and stress during COVID-19 pandemic
Source: PLoS One. 2022 Mar 23;17(3):e0265733. doi: 10.1371/journal.pone.0265733 (PMC8942203; doi:10.1371/journal.pone.0265733)
Supplement: S3 File — (PDF) [file pone.0265733.s003.pdf]

PERCEIVED STRESS SCALE (PSS-10)

The questions in this scale ask you about your feelings and thoughts during the **last month**.

In each case, you will be asked to indicate by circling how often you felt or thought a certain way.

0 = Never 1 = Almost Never 2 = Sometimes 3 = Fairly Often 4 = Very Often

1. In the last month, how often have you been upset because of something that happened unexpectedly?	0	1	2	3	4
2. In the last month, how often have you felt that you were unable to control the important things in your life?	0	1	2	3	4
3. In the last month, how often have you felt nervous and "stressed"?	0	1	2	3	4
4. In the last month, how often have you felt confident about your ability to handle your personal problems?	0	1	2	3	4
5. In the last month, how often have you felt that things were going your way?	0	1	2	3	4
6. In the last month, how often have you found that you could not cope with all the things that you had to do?	0	1	2	3	4
7. In the last month, how often have you been able to control irritations in your life?	0	1	2	3	4
8. In the last month, how often have you felt that you were on top of things?	0	1	2	3	4
9. In the last month, how often have you been angered because of things that were outside of your control?	0	1	2	3	4
10. In the last month, how often have you felt difficulties were piling up so high that you could not overcome them?	0	1	2	3	4

References: Cohen, S. and Williamson, G. Perceived Stress in a Probability Sample of the United States. Spacapan, S. and Oskamp, S. (Eds.) The Social Psychology of Health. Newbury Park, CA: Sage, 1988.
