# Supplementary material for: Prevalence of symptomatic dry eye disease with associated risk factors among medical students at Chiang Mai University due to increased screen time and stress during COVID-19 pandemic
Source: PLoS One. 2022 Mar 23;17(3):e0265733. doi: 10.1371/journal.pone.0265733 (PMC8942203; doi:10.1371/journal.pone.0265733)
Supplement: S4 File — (PDF) [file pone.0265733.s004.pdf]

แบบวัดความรู้สึกเครียด

Thai version of 10-Item Perceived Stress Scale-10 (T-PSS-10)

คำแนะนำ: ต่อไปนี้เป็นคำถามเกี่ยวกับความรู้สึกและความคิดของท่านในรอบ 1 เดือนที่ผ่านมา โปรดกากบาท X ในช่อง ❑ ที่ตรงกับที่ท่านคิดหรือรู้สึกแบบนั้น	ไม่เลย<br>0	แทบจะไม่มี<br>1	มีบางครั้ง<br>2	ค่อนข้างบ่อย<br>3	บ่อยมาก<br>4
1. ในรอบ 1 เดือนที่ผ่านมาบ่อยแค่ไหนที่ท่าน รู้สึกไม่สบายใจเพราะมีสิ่งที่เกิดขึ้นอย่างไม่ดาดคิด?	❑	❑	❑	❑	❑
2. ในรอบ 1 เดือนที่ผ่านมาบ่อยแค่ไหนที่ท่านรู้สึกว่าท่านไม่สามารถควบคุมเรื่องสำคัญ ๆ ในชีวิตของท่านได้?	❑	❑	❑	❑	❑
3. ในรอบ 1 เดือนที่ผ่านมา บ่อยแค่ไหนที่ท่านรู้สึกกระสับกระส่ายและตึงเครียด?	❑	❑	❑	❑	❑
4. ในรอบ 1 เดือนที่ผ่านมา บ่อยแค่ไหนที่ท่านรู้สึกมั่นใจในความสามารถของตนเองที่จะรับมือกับปัญหาส่วนตัวทั้งหลายได้?	❑	❑	❑	❑	❑
5. ในรอบ 1 เดือนที่ผ่านมา บ่อยแค่ไหนที่ท่านรู้สึกว่าสิ่งทั้งหลายเป็นไปในทิศทางที่ท่านต้องการ?	❑	❑	❑	❑	❑
6. ในรอบ 1 เดือนที่ผ่านมา บ่อยแค่ไหนที่ท่านรู้สึกว่าท่านไม่สามารถจัดการกับสิ่งทั้งหลายทั้งที่เป็นสิ่งที่ล้วนเคยทำมาแล้ว?	❑	❑	❑	❑	❑
7. ในรอบ 1 เดือนที่ผ่านมา บ่อยแค่ไหนที่ท่านรู้สึกว่าสามารถควบคุมสิ่งทั้งหลายที่มากวนใจได้?	❑	❑	❑	❑	❑
8. ในรอบ 1 เดือนที่ผ่านมา บ่อยแค่ไหนที่ท่านรู้สึกว่าท่านควบคุมสถานการณ์ต่าง ๆ ได้?	❑	❑	❑	❑	❑
9. ในรอบ 1 เดือนที่ผ่านมา บ่อยแค่ไหนที่ท่านโกรธอันเนื่องมาจากสิ่งที่นอกเหนือการควบคุมของท่าน?	❑	❑	❑	❑	❑
10.ในรอบ 1 เดือนที่ผ่านมา บ่อยแค่ไหนที่ท่าน รู้สึกว่าปัญหาต่าง ๆ ทับถมมากขึ้นจนท่านไม่สามารถแก้ไขได้หมด?	❑	❑	❑	❑	❑

เฉพาะเจ้าหน้าที่ ❑❑/40

Wongpakaran N, Wongpakaran T. The Thai version of the PSS-10: An Investigation of its psychometric properties. Biopsychosoc Med. 2010;4:6.

สงวนลิขสิทธิ์
