# Supplementary material for: Prevalence of symptomatic dry eye disease with associated risk factors among medical students at Chiang Mai University due to increased screen time and stress during COVID-19 pandemic
Source: PLoS One. 2022 Mar 23;17(3):e0265733. doi: 10.1371/journal.pone.0265733 (PMC8942203; doi:10.1371/journal.pone.0265733)
Supplement: S6 File — (PDF) [file pone.0265733.s006.pdf]

แบบสอบถาม Ocular surface Index Score (OSDI)

แบบสอบถามนี้ประกอบด้วยคำถาม 12 ข้อ
ให้วงกลมตัวเลขที่อยู่ในกรอบสี่เหลี่ยมในแต่ละข้อตามคำตอบของผู้ตอบแบบสอบถาม
หลังจากนั้นให้กรอกตัวเลขลงในกรอบสี่เหลี่ยม A, B, C, D และ E ตามคำแนะนำด้านล่าง

■ ใน 1 สัปดาห์ที่ผ่านมา ท่านเคยมีอาการต่อไปนี้หรือไม่ ?

	มีอาการ ตลอดเวลา	มีอาการ เป็นส่วนใหญ่	มีอาการเป็น ประมาณครึ่งหนึ่ง	มีอาการ เป็นบางครั้ง	ไม่มีอาการเลย
1. ตาสู้แสงจ้าไม่ได้	4	3	2	1	0
2. รู้สึกฝืดตา กระพริบตาลำบาก	4	3	2	1	0
3. ปวดตาหรือเจ็บตา	4	3	2	1	0
4. มองเห็นภาพเบลอ	4	3	2	1	0
5. การมองเห็นแย่ลง	4	3	2	1	0

ผลรวมของคะแนนข้อ 1-5 (A)

■ ใน 1 สัปดาห์ที่ผ่านมา อาการข้างต้นมีผลกระทบต่อกิจกรรมเหล่านี้หรือไม่ ?

	มีผลกระทบ ตลอดเวลา	มีผลกระทบ เป็นส่วนใหญ่	มีผลกระทบ ประมาณครึ่งหนึ่ง	มีผลกระทบ เป็นบางครั้ง	ไม่มีผล กระทบเลย	
6. อ่านหนังสือ	4	3	2	1	0	ไม่ตอบ
7. ขับรถในเวลากลางคืน	4	3	2	1	0	ไม่ตอบ
8. มองหน้าจอคอมพิวเตอร์หรือหน้าจอ ATM	4	3	2	1	0	ไม่ตอบ
9. ดูโทรทัศน์	4	3	2	1	0	ไม่ตอบ

ผลรวมของคะแนนข้อ 6-9 (B)

■ ใน 1 สัปดาห์ที่ผ่านมา ท่านรู้สึกไม่สบายตาในสถานการณ์เหล่านี้หรือไม่ ?

	ไม่สบายตา ตลอดเวลา	ไม่สบายตา เป็นส่วนใหญ่	ไม่สบายตา ประมาณครึ่งหนึ่ง	ไม่สบายตา เป็นบางครั้ง	ไม่มีอาการเลย	
10. เมื่อมีลมพัดแรง	4	3	2	1	0	ไม่ตอบ
11. เมื่ออยู่ในสถานที่มีความชื้นต่ำ	4	3	2	1	0	ไม่ตอบ
12. เมื่ออยู่ในห้องปรับอากาศ	4	3	2	1	0	ไม่ตอบ

ผลรวมของคะแนนข้อ 10-12 (C)

ผลรวมของคะแนนในช่อง A, B และ C (D)

จำนวนคำถามที่ได้รับคำตอบ (E)
