# Supplementary material for: Prevalence of symptomatic dry eye disease with associated risk factors among medical students at Chiang Mai University due to increased screen time and stress during COVID-19 pandemic
Source: PLoS One. 2022 Mar 23;17(3):e0265733. doi: 10.1371/journal.pone.0265733 (PMC8942203; doi:10.1371/journal.pone.0265733)
Supplement: Attachment — Submitted filename: Responses to Reviewer_20210711 CT edit.docx [file pone.0265733.s007.docx]

Dear Editor in Chief and all reviewers

Journal of PLoS ONE

Revision of submitted manuscript: [PONE-D-21-18381] - [EMID:7fa3bce5a1aded7e]

**Prevalence of symptomatic dry eye disease with associated risk factors among medical students at Chiang Mai University during COVID-19 pandemic**

We do appreciate your valuable time reviewing our manuscript for publication with revision suggestions. So, we would like to submit our thoroughly point-by-point response (blue fonts) to your reviews (black fonts), as stated in the letter of Jun 26, 2021, as follows:

**Part I: Journal Requirements:**

When submitting your revision, we need you to address these additional requirements.

1. Please ensure that your manuscript meets PLOS ONE's style requirements, including those for file naming.

-The manuscript format is reviewed and meets PLOS ONE's style requirements.

2. Thank you for stating the following in the Funding Section of your manuscript:

"This research was funded by Faculty of Medicine, Chiang Mai University, Chiang Mai, Thailand
(Grant number: 043/2564). The funder has no role in the study design, data collection and analysis, decision to publish, or preparation of the manuscript."

We note that you have provided funding information that is not currently declared in your Funding Statement. However, funding information should not appear in the Acknowledgments section or other areas of your manuscript. We will only publish funding information present in the Funding Statement section of the online submission form. 
Please remove any funding-related text from the manuscript and let us know how you would like to update your Funding Statement. Currently, your Funding Statement reads as follows:

"This research was funded by Faculty of Medicine, Chiang Mai University, Chiang Mai, Thailand (Grant number: 043/2564). The funder has no role in the study design, data collection and analysis, decision to publish, or preparation of the manuscript. "

Please include your amended statements within your cover letter; we will change the online submission form on your behalf.

- The funding information has been removed from the Acknowledgments section. There has been no change with the current Funding Statement. Please kindly change the online submission form on our behalf.

"This research was funded by Faculty of Medicine, Chiang Mai University, Chiang Mai, Thailand (Grant number: 043/2564). The funder has no role in the study design, data collection and analysis, decision to publish, or preparation of the manuscript. "

3. In your Data Availability statement, you have not specified where the minimal data set underlying the results described in your manuscript can be found. PLOS defines a study's minimal data set as the underlying data used to reach the conclusions drawn in the manuscript and any additional data required to replicate the reported study findings in their entirety. All PLOS journals require that the minimal data set be made fully available.

"Upon re-submitting your revised manuscript, please upload your study’s minimal underlying data set as either Supporting Information files or to a stable, public repository and include the relevant URLs, DOIs, or accession numbers within your revised cover letter.

Important: If there are ethical or legal restrictions to sharing your data publicly, please explain these restrictions in detail.

We will update your Data Availability statement to reflect the information you provide in your cover letter.

- The information of Availability of Data and Materials has been added as follows:

The datasets used and/or analyzed in this study are available from the corresponding author on reasonable request.

4. We note that Figures 1 and 2 in your submission contain copyrighted images. All PLOS content is published under the Creative Commons Attribution License (CC BY 4.0), which means that the manuscript, images, and Supporting Information files will be freely available online, and any third party is permitted to access, download, copy, distribute, and use these materials in any way, even commercially, with proper attribution.
 
We require you to either (1) present written permission from the copyright holder to publish these figures specifically under the CC BY 4.0 license, or (2) remove the figures from your submission:
 
a. You may seek permission from the original copyright holder of Figures 1 and 2 to publish the content specifically under the CC BY 4.0 license. 
 
We recommend that you contact the original copyright holder with the Content Permission Form. Please be aware that this license allows unrestricted use and distribution, even commercially, by third parties. Please reply and provide explicit written permission to publish XXX under a CC BY license and complete the attached form.”
 
Please upload the completed Content Permission Form or other proof of granted permissions as an "Other" file with your submission. 
 
In the figure caption of the copyrighted figure, please include the following text: “Reprinted from [ref] under a CC BY license, with permission from [name of publisher], original copyright [original copyright year].”

b. If you are unable to obtain permission from the original copyright holder to publish these figures under the CC BY 4.0 license or if the copyright holder’s requirements are incompatible with the CC BY 4.0 license, please either i) remove the figure or ii) supply a replacement figure that complies with the CC BY 4.0 license. Please check copyright information on all replacement figures and update the figure caption with source information. If applicable, please specify in the figure caption text when a figure is similar but not identical to the original image and is therefore for illustrative purposes only.

- All images in Figures 1 and 2 were obtained from participants in this study with informed consent. Moreover, during revision, we have added more photographic techniques (Reflected IR, Trans IR) with references. (21 and 22)

21.	Adil MY, Xiao J, Olafsson J, Chen X, Lagali NS, Ræder S, et al. Meibomian Gland Morphology Is a Sensitive Early Indicator of Meibomian Gland Dysfunction. Am J Ophthalmol. 2019;200:16-25. Epub 2018/12/24. doi: 10.1016/j.ajo.2018.12.006. PubMed PMID: 30578784.

22.	Halleran C, Kwan J, Hom M, Harthan J, editors. Agreement in reading centre grading of meibomian gland tortuosity and atrophy. Poster presented at American Academy of Optometry annual meeting: November; 2016.

And, the additional figure legends for Figure 1 and 2 as follows:

**Fig 1. The Meibograde grading system: subjective grading of meibomian gland loss.** Row A: Dynamic illumination mode (Reflected infrared) of upper lids; Row B: Dynamic illumination mode (Reflected infrared) of lower lids; Row C: Adaptive transillumination mode (Trans infrared) of lower lids.

**Fig 2. The Meibomian gland tortuosity grading system.** Row A: Dynamic illumination mode (Reflected infrared) of upper lids; Row B: Dynamic illumination mode (Reflected infrared) of lower lids; Row C: Adaptive transillumination mode (Trans infrared) of lower lids; Row D: Dual mode (combined dynamic illumination and adaptive transillumination) of lower lids.

**Part II: Reviewers' comments:**

Please use the space provided to explain your answers to the questions above. You may also include additional comments for the author, including concerns about dual publication, research ethics, or publication ethics. (Please upload your review as an attachment if it exceeds 20,000 characters)

 Reviewer #1: Chulaluck et al. describe a prospective cross-sectional study aimed to explore the prevalence of symptomatic Dry eye disease (DED) with associated risk factors among medical students at Chiang Mai University during covid-19 pandemic
Comments:
1. Abstract: The study did not include any clinical evaluation of the participants; therefore some of them could potentially have other ocular conditions that may have caused dry eye symptoms.

- The study focused on the dry eye symptoms evaluating by the questionnaires and the meibomian gland function evaluating by the LipiView® Interferometer.

2. The use of a surgical face mask – which is known to increase dry eye symptoms especially during the COVID-19 pandemic, was not part of the risk factors taken in consideration.

- Thank you for your comment. All students are always wearing a face mask during social activities. Therefore, this factor was equally distributed between groups and has not been included in the analysis.

3. The information collected during the study only represented the participants' condition at the day of their examination.

- Thought the data was collected on one day. However, the questionnaires represented general symptoms during a recent period; moreover, the meibomian gland function does not alter rapidly without any medical interventions. So, our data could effectively describe the prevalence of symptomatic dry eye disease with associated risk factors among medical students at Chiang Mai University during COVID-19 pandemic.

4. Methods section: was not elaborate enough. Exclusion and inclusion criteria are not detailed enough.

- More details of exclusion and inclusion criteria were added in the materials and methods section as follows:

“Only medical students who completed the questionnaire and the LipiView® II interferometer examination were included in the analysis. All participants can refuse to be in the study at all, or to stop participating at any time of the study.”

And

“Participants who had a history of ocular surgery or trauma within 3 months, or ocular diseases such as ocular infection, allergy, autoimmune disease, and those using punctal plug or topical ocular medications other than artificial tears were excluded.”

5. The LipiView test is designed to measure the thickness of the lipid layer, but recent studies showed that It doesn’t seem to correlate too well with people’s slit lamp exams or symptoms.

- In our study, lipid thickness was considered as one factor derived from meibomian gland evaluation. However, in the univariate analysis, the lipid thickness was not significantly different among groups of participants with and without dry eye symptoms.

6. The study only included medical students from Chiang Mai University, which may not represent all medical students in Thailand.

- Thank you for your comment. Though our study included medical students from a single university in Thailand, we believe that our data was useful and could represent the general characteristics of dry eye symptoms of medical students in Thailand for two main reasons. First, our study included a large number of medical students (N = 528). Secondly, we believe that the characteristics of medical students in different parts of Thailand should not be markedly different.

Reviewer #2: We would like to thank the authors for their work on the Prevalence of symptomatic Dry eye disease with associated risk factors among medical students at Chiang Mai University during COVID-19 pandemic. We think it is a well written paper.

We still would like to give some advice on correction:

1.) Please correct the punctuations. There are lots of mistakes regarding the commas options

- The manuscript was revised by a native American speaker. However, we are willing to revise the manuscript again once all corrections are done regarding the English correction.

Introduction:
2.) Please change “The prevalence of dry eye disease (DED) in Asians is higher than Caucasians.” To “The prevalence of dry eye disease (DED) in Asians is higher than in Caucasians. “ and name a reference

- Thank you for your comment. The correction has been done. However, the references for this statement were of number 5 and 6 as follows:

5.	Stapleton F, Alves M, Bunya VY, Jalbert I, Lekhanont K, Malet F, et al. TFOS DEWS II Epidemiology Report. The ocular surface. 2017;15(3):334-65. Epub 2017/07/25. doi: 10.1016/j.jtos.2017.05.003. PubMed PMID: 28736337.

6.	Lekhanont K, Rojanaporn D, Chuck RS, Vongthongsri A. Prevalence of dry eye in Bangkok, Thailand. Cornea. 2006;25(10):1162-7. Epub 2006/12/19. doi: 10.1097/01.ico.0000244875.92879.1a. PubMed PMID: 17172891.

3.) “The international workshop defined MGD as a chronic, diffuse abnormality of the meibomian glands, usually characterized by an obstruction of terminal duct and/or changes in the secretion of the glands qualitatively/quantitatively. Please name a reference

- Thank you for your comment. The reference for this definition was in number 7 as follow:

7.	Nelson JD, Shimazaki J, Benitez-del-Castillo JM, Craig JP, McCulley JP, Den S, et al. The international workshop on meibomian gland dysfunction: report of the definition and classification subcommittee. Invest Ophthalmol Vis Sci. 2011;52(4):1930-7. Epub 2011/04/01. doi: 10.1167/iovs.10-6997b. PubMed PMID: 21450914; PubMed Central PMCID: PMCPMC3072158.

And the reference has already been mentioned in the following paragraph.

“The international workshop defined MGD as a chronic, diffuse abnormality of the meibomian glands, usually characterized by an obstruction of terminal duct and/or changes in the secretion of the glands qualitatively/quantitatively. The condition may lead to a tear film alteration, symptoms of eye irritation and inflammation, and ocular surface disease [7].”

4.) The survey questionnaire had four sections: demographic data with pre-existing medical conditions; risk factors for DED including personal habits; psychological stress; and a dry eye questionnaire using the Ocular Surface Disease Index (OSDI), please attach the questionnaire

- Thank you for your comment. The questionnaire will be added as a supplemental in both Thai and English versions.

5.) Please change “Participants who used artificial tears were instructed to stop using for 6 hours before LipiView® II examination. “ to “Participants who used artificial tears were instructed to stop the use 6 hours before LipiView® II examination.”

- Thank you for your comment. The change has been done as suggested.

LipiView® II Ocular Surface interferometer:
6.) “For the blinking pattern, the incomplete blinking ratio was calculated by the number of incomplete blinks divided by the total blinks.” Please refer to the attached picture

- Blinking pattern is used to assess the quality of blinking. The best quality of blinking pattern is that every blink is complete (defined as the complete apposition of upper and lower lids.). The LipiView could demonstrate the number of incomplete and complete blinks per 20 seconds and also calculate the partial blinking rate (PBR). The reference 20 is added. However, there was no figure for the blinking pattern.

20.	Eom Y, Lee JS, Kang SY, Kim HM, Song JS. Correlation between quantitative measurements of tear film lipid layer thickness and meibomian gland loss in patients with obstructive meibomian gland dysfunction and normal controls. Am J Ophthalmol. 2013;155(6):1104-10.e2. Epub 2013/03/08. doi: 10.1016/j.ajo.2013.01.008. PubMed PMID: 23465270.

Results:
7.) 392/528 is 74,24% not 85%

- Thank you for your comment. The change has been done as suggested. (in Results)

8.) 69/528 is 13% not 15%

- Thank you for your comment. The change has been done as suggested. (in Results)

Table 1:
9.)
10.) Please change “average” to “average”

- The change has been done as suggested. (in Table 1)

Discussion:
11.) “Hyon et al.“ Please also name the reference here

- Thank you for your comment. The reference for this definition was in number 15 as follow:

15.	Hyon JY, Yang HK, Han SB. Dry Eye Symptoms May Have Association With Psychological Stress in Medical Students. Eye & contact lens. 2019;45(5):310-4. Epub 2018/12/27. doi: 10.1097/icl.0000000000000567. PubMed PMID: 30585856.

And the reference has already been mentioned in the following paragraph.

“Hyon *et al*. demonstrated that stress, female sex, contact lens wear, and duration of using VDT were significant risk factors for DED. The prevalence of DED based on the symptoms in Korean medical students was 27.1% [15].”

12.) “Health promotion with the “20-20-20” rule and limited screen time may be very helpful.“ Please explain this rule

- Thank you for your comment. The explanation has been added as suggested.

“Health promotion with the “20-20-20” rule, which recommends that every 20 minutes, an individual should take a 20-second break and focus their eyes on something at least 20 feet away. and limited screen time may be very helpful.”

Reviewer #3: Dear author,
Thank you for you submission.
There are some points to consider:
1. The title of the article says that the DED was diagnosed during the COVID-19 pandemic, and the discussion mention that during the pandemic medical students were prone to more stress (by T-PSS-10) and VDT time. There weren’t any comparisons of these variables before and during the pandemic. Moreover, do you have any evidence that the DED symptoms / signs were more severe during the pandemic in comparison to the time before?

- Thank you for your comment. As the study was conducted for the first time during the pandemic, there has been no previous report on the prevalence of dry eye symptoms in this specific population. However, regarding the previous study of the prevalence of dry eye in Thai adults which reported to be 34% [5], the prevalence of dry eye in our study was higher.

2. The article concludes that the medical students in Chiang may had a higher rate of DED comparing other studies. You chose the COVID-19 pandemic as one possible reason. Besides the previous note, some other genetic or environmental factors should be considered.

- Thank you for your comment. Please allow us to clarify the COVID-19 pandemic as a possible reason of higher prevalence of dry eye symptoms. In our study, the COVID-19 pandemic was taken into consideration as the pandemic leads to both behavioral and environmental changes that may aggravate the dry eye symptoms. For example, during the pandemic, most of lectures were conducted via the online programmes which lead to more screen time. Also, the circumstances may stress all the medical students in many ways as discussed in the manuscript. In our study, the genetic factors, though might be significant, but was not included.

3. The prevalence of DED was based on OSDI score of >12 and the prevalence was 70.8% which is higher than other articles. However, the articles that were cited used other methods or in combination with OSDI score for diagnosing DED. Therefore it should be mentioned as a limitation.

- Thank you for your comment. The following statement has been added to the limitation part.

“Moreover, the prevalence of DED may be varied from the different diagnostic methods.”

4. In the discussion you mention female sex was a risk factor for DED, but in the regression analysis (table 3) it was not significant.

- Thank you for your comment. That is correct that in the regression analysis, the p value for female sex was 0.052. Therefore, we stated that female sex was likely to be associated with an increased risk of DED, similar to the previous studies.

5. Another limitation that should be mentioned is that only one observer assessed the Meibomian glands. Did the observer assessed every eyelid separately? Or did he/she assessed all 4 eyelids as one unit knowing that they belong to the same patient? The second option could lead to an observer bias.

- Thank you for your comment. In this study, a single experienced observer (CT) subjectively evaluated the meibomian gland dropouts in both upper and lower eyelids using a validated Meibograde grading scheme. Each eyelid was blinded for evaluation and the upper and lower lids were separately evaluated.

We appreciate for the very helpful review.

Sincerely Yours,

Chulaluck Tangmonkongvoragul, MD

Department of Ophthalmology,

Faculty of Medicine,

Chiang Mai University,

Chiang Mai, Thailand

E-mail: poupae025@gmail.com, chulaluck.t@cmu.ac.th
