# Supplementary material for: Prevalence of symptomatic dry eye disease with associated risk factors among medical students at Chiang Mai University due to increased screen time and stress during COVID-19 pandemic
Source: PLoS One. 2022 Mar 23;17(3):e0265733. doi: 10.1371/journal.pone.0265733 (PMC8942203; doi:10.1371/journal.pone.0265733)
Supplement: Attachment — Submitted filename: Responses to Reviewer_20210826.docx [file pone.0265733.s008.docx]

Dear Editor in Chief and all reviewers

Journal of PLoS ONE

Revision of submitted manuscript: [PONE-D-21-18381R1]

**Prevalence of symptomatic dry eye disease with associated risk factors among medical students at Chiang Mai University during COVID-19 pandemic**

We do appreciate your valuable time reviewing our manuscript for publication with revision suggestions. So, we would like to submit our thoroughly point-by-point response (blue fonts) to your reviews (black fonts), as stated in the letter of Aug 24, 2021, as follows:

6. Review Comments to the Author

Reviewer #3: Dear author, Thank you for your response. There are still issues that need to be addressed correctly:

1. Explaining the prevalence of DED by “the pandemic” is not correct, you should evaluate each factor separately. Please add references for comparison of the following factors before and after the pandemic – dry eye prevalence in similar age group (comparing to adults is not sufficient); students’ psychological stress; students’ computer and screen time use.

- Thank you for your comment. In our study, the pandemic is not directly a risk factor for DED in medical students, but rather be a unique situation that interfered with the known risk factors for DED. We believe that during the pandemic, medical students had to dramatically change the ways of learning and also their lifestyles which might affect with the DED symptoms. However, due to limited number of the DED prevalence in a specific population like medical students, the comparison group as general adult population might be sufficient, and further studies of the DED prevalence in medical students in Thailand when the pandemic is subsided, is an interesting idea for our future project.

2. If every eyelid was evaluated separately by the blinded single observer, this should be noted.

-Thank you for your comment. The following statement has been added to the methods part.

A single experienced observer (CT) subjectively evaluated the meibomian gland dropout in both upper and lower eyelids using a validated Meibograde grading scheme, with a 4-point scale from 0 to 3 in which grade 0 is 0-25% meibomian gland loss; grade 1, 26-50% loss; grade 2, 51-75% loss; and grade 3, more than 75% loss [21]. The meibomian gland loss was calculated with reference to the equivalent meibomian gland area in healthy individuals. Meibomian gland tortuosity for each eyelid was graded using the 5-point Halleran scale: grade 0, no tortuosity; grade 1, less than 25% tortuosity; grade 2, 26-50% tortuosity; grade 3, 51-74% tortuosity; and grade 4, more than 75% tortuosity [22]. Each eyelid was blinded for evaluation and the upper and lower lids were separately evaluated.

We appreciate for the very helpful review.

Sincerely Yours,

Chulaluck Tangmonkongvoragul, MD

Department of Ophthalmology,

Faculty of Medicine,

Chiang Mai University,

Chiang Mai, Thailand

E-mail: poupae025@gmail.com, chulaluck.t@cmu.ac.th
