# Supplementary material for: Prevalence of symptomatic dry eye disease with associated risk factors among medical students at Chiang Mai University due to increased screen time and stress during COVID-19 pandemic
Source: PLoS One. 2022 Mar 23;17(3):e0265733. doi: 10.1371/journal.pone.0265733 (PMC8942203; doi:10.1371/journal.pone.0265733)
Supplement: Attachment — Submitted filename: Responses to Reviewer_20211210.docx [file pone.0265733.s009.docx]

Dear Editor in Chief and all reviewers

Journal of PLoS ONE

Revision of submitted manuscript: [PONE-D-21-18381R2]

**Prevalence of symptomatic dry eye disease with associated risk factors among medical students at Chiang Mai University during COVID-19 pandemic**

We do appreciate your valuable time reviewing our manuscript for publication with revision suggestions. So, we would like to submit our thoroughly point-by-point response (blue fonts) to your reviews (black fonts), as stated in the letter of Nov 16, 2021, as follows:

**6. Review Comments to the Author**

**Reviewer #2:** We do thank the authors for addressing our comments. We still have one point to address. We think having an adequate control group is essential. Please look for better comparison to make your data valid. Please look for DED in a more comparable group like other students before the pandemic.

Compare the risk factors for DED one by one before and after the pandemic like "psychological stress", screen and computer time.

- Thank you for your comment. We have added the findings of Hyon *et al*. study which conducted the study of DED in Asian medical students before the COVID-19 pandemics in the second paragraph of the discussion part as follows:

Hyon *et al*. demonstrated that stress, female sex, contact lens wear, and duration of using VDT were significant risk factors for DED. The prevalence of DED based on the symptoms in Korean medical students was 27.1%. This study evaluated DED in medical students before the COVID-19 pandemics and revealed that DED may have association with psychological stress (using the Perceived Stress Scale 4 (PSS-4) questionnaire) [15]. Additionally, the PSS scores in our study tended to be higher than the study by Hyon *et al*., though the direct comparison was not allowed due to the different versions used. Therefore, a higher prevalence of symptomatic DED in medical students (70.8%) in our study may be explained by more stressful situation during the COVID-19 pandemic among medical students.

We appreciate for the very helpful review.

Sincerely Yours,

Chulaluck Tangmonkongvoragul, MD

Department of Ophthalmology,

Faculty of Medicine,

Chiang Mai University,

Chiang Mai, Thailand

E-mail: poupae025@gmail.com, chulaluck.t@cmu.ac.th
