# Supplementary material for: Prevalence of symptomatic dry eye disease with associated risk factors among medical students at Chiang Mai University due to increased screen time and stress during COVID-19 pandemic
Source: PLoS One. 2022 Mar 23;17(3):e0265733. doi: 10.1371/journal.pone.0265733 (PMC8942203; doi:10.1371/journal.pone.0265733)
Supplement: Attachment — Submitted filename: Responses to Reviewers_7 Feb 2022.docx [file pone.0265733.s010.docx]

Dear Editor in Chief and all reviewers

Journal of PLOS ONE

Revision of submitted manuscript: [PONE-D-21-18381R3]

**Prevalence of symptomatic dry eye disease with associated risk factors among medical students at Chiang Mai University during COVID-19 pandemic**

We do appreciate your valuable time reviewing our manuscript for publication with revision suggestions. So, we would like to submit our thoroughly point-by-point response (blue fonts) to your reviews (black fonts), as stated in the letter of Jan 24, 2022, as follows:

**6. Review Comments to the Author**

**Reviewer #3:** Thank you for you submission.
Because it may be somehow misleading, I recommend the editor to reconsider the title of the article "during COVID-19 pandemic" as there are no direct causes for dry eye due to COVID-19 as the manuscript suggest, but only indirect risk factors (screen time, face masks).
all other notes has been addressed.

- Thank you for your comment. We have changed the title of the article to avoid misleading as follows:

Prevalence of symptomatic dry eye disease with associated risk factors among medical students at Chiang Mai University due to increased screen time and stress during COVID-19 pandemic

We appreciate for the very helpful review.

Sincerely Yours,

Chulaluck Tangmonkongvoragul, MD

Department of Ophthalmology,

Faculty of Medicine,

Chiang Mai University,

Chiang Mai, Thailand

E-mail: poupae025@gmail.com, chulaluck.t@cmu.ac.th
